# Chromatin accessibility and transcriptome integrative analysis revealed AP-1-mediated genes potentially modulate histopathology features in psoriasis

**DOI:** 10.1186/s13148-022-01250-6

**Published:** 2022-03-11

**Authors:** Xiaoqing Xu, Xianfa Tang, Yuxi Zhang, Zhaobing Pan, Qingping Wang, Lili Tang, Caihong Zhu, Hui Cheng, Fusheng Zhou

**Affiliations:** 1 Department of Dermatology, The First Affiliated Hospital, Anhui Medical University, Hefei, 230032 Anhui China; 2 Institute of Dermatology, Anhui Medical University, Hefei, 230032 Anhui China; 3 Key Laboratory of Dermatology (Anhui Medical University), Ministry of Education, Hefei, 230032 Anhui China; 4 Inflammation and Immune Mediated Diseases Laboratory of Anhui Province, Hefei, 230032 Anhui China

**Keywords:** Psoriasis, AP-1, BETA, ATAC-seq, Integrated analysis, Histopathology, Gene expression

## Abstract

**Background:**

Psoriasis is a chronic and hyperproliferative skin disease featured by hyperkeratosis with parakeratosis, Munro micro-abscess, elongation of rete pegs, granulosa thinning, and lymphocyte infiltration. We previously profiled gene expression and chromatin accessibility of psoriatic skins by transcriptome sequencing and ATAC-seq. However, integrating both of these datasets to unravel gene expression regulation is lacking. Here, we integrated transcriptome and ATAC-seq of the same psoriatic and normal skin tissues, trying to leverage the potential role of chromatin accessibility and their function in histopathology features.

**Results:**

By inducing binding and expression target analysis (BETA) algorithms, we explored the target prediction of transcription factors binding in 15 psoriatic and 19 control skins. BETA identified 408 upregulated genes (rank product < 0.01) and 133 downregulated genes linked with chromatin accessibility. We noticed that cumulative fraction of genes in upregulation group was statistically higher than background, while that of genes in downregulation group was not significant. KEGG pathway analysis showed that the upregulated 408 genes were enriched in TNF, NOD, and IL-17 signaling pathways. In addition, the motif module in BETA suggested the 57 upregulated genes are targeted by transcription factor AP-1, indicating that increased chromatin accessibility facilitated the binding of AP-1 to the target regions and further induced expression of relevant genes. Among these genes, SQLE, STRN, EIF4, and MYO1B expression was increased in patients with hyperkeratosis, parakeratosis, and acanthosis thickening.

**Conclusions:**

In summary, with the advantage of BETA, we identified a series of genes that contribute to the disease pathogenesis, especially in modulating histopathology features, providing us with new clues in treating psoriasis.

**Supplementary Information:**

The online version contains supplementary material available at 10.1186/s13148-022-01250-6.

## Background

Psoriasis is an immune-mediated multigenic skin disease characterized by symmetrically well-defined erythema, covered with silvery scales involving elbows, knees, torso, and scalp [1]. The histopathological traits of psoriasis are diverse, typically including hyperkeratosis with parakeratosis and immune cell infiltration, Munro micro-abscess, acanthosis thickening, vascular dilatation congestion, elongation of rete pegs, and granulosa thinning. Psoriasis patients are usually accompanied by mental and physical burden because of its high incidence, chronic course, disability, malformation, and comorbidities, such as metabolic syndrome and cardiovascular diseases [2, 3]. To address this burden, scientists have tried to unravel the etiology and pathogenesis of psoriasis with various strategies and have made significant progress, but there are still many mysteries. More than 80 susceptibility genes were recently identified [4], and some cytokines, such as TNF-α, IL-17, and IL-23, have been developed for biological agents [5]. However, the exact pathogenesis of psoriasis was still not fully revealed.

The histopathological features of psoriasis indicate a critical alteration in disease progression, but the molecular mechanism under these features is largely unknown. Few studies aimed to link epigenetic modifications with the histopathological characteristics in psoriasis. For example, Chandra et al. carried out genome-wide DNA methylation to figure out which epigenetic loci are associated with Munro micro-abscess in psoriasis [6]. Nevertheless, chromatin accessibility and its potential regulatory roles in histopathological changes are still missing.

Assay for targeting accessible-chromatin with high-throughput sequencing (ATAC-seq), based on Tn5 transposase hyperactivity, helps us investigate genome chromatin accessibility and reveals multiple aspects of transcriptional regulation [7]. It provides the whole open chromatin across the entire genome at one time, exploring transcription factor binding and gene expression regulation, which has been widely used in various diseases [8], including psoriasis. Utilizing ATAC-seq on 15 psoriatic lesions, 9 non-psoriatic lesions, and 19 normal healthy skin tissues, we previously identified 4,195 differentially accessible regions [9]. Further analysis showed that the sequence of differentially accessible regions was enriched in the FRA1/AP-1 transcription factor binding region [9]. Upregulation of AP-1 family members has been shown in psoriatic skins, but the exact mechanism is not precise [10]. Currently, we tried to perform an integrative analysis of RNA-seq and ATAC-seq data, exploring the potential network of AP-1 regulating psoriasis and aiming to find out whether some genes are implicated in histopathological alterations.

Several methods have been used to integrate transcription and chromatin accessibility datasets by directly overlapping relevant genes, which might underestimate the potential roles of TF binding targets [7, 11–13]. The BETA algorithm is an efficient web tool to identify motifs of transcription factors, infer their target genes, and explore these factors' activating or repressive status [14]. To search the direct targets of differentially accessible regions, we attempted to perform BETA-plus (Version 1.0.7) to integrate ATAC-seq accessible peak data with differential expression data. Interestingly, we identified 408 upregulated genes and 133 downregulated genes (rank product < 0.01) and depicted significant binding motifs and putative collaborating factors. These upregulated genes were strongly targeted by AP-1 family transcription factors. Then, we found that their gene expression differences were related to the different pathological manifestations of psoriasis. It provides us with a novel insight into the potential regulatory mechanism and therapy of psoriasis and a future direction for us to deeply explain the mechanism of AP-1 in psoriatic lesions.

## Results

### The prediction of TFs' function and direct targets

**Fig. 1:**
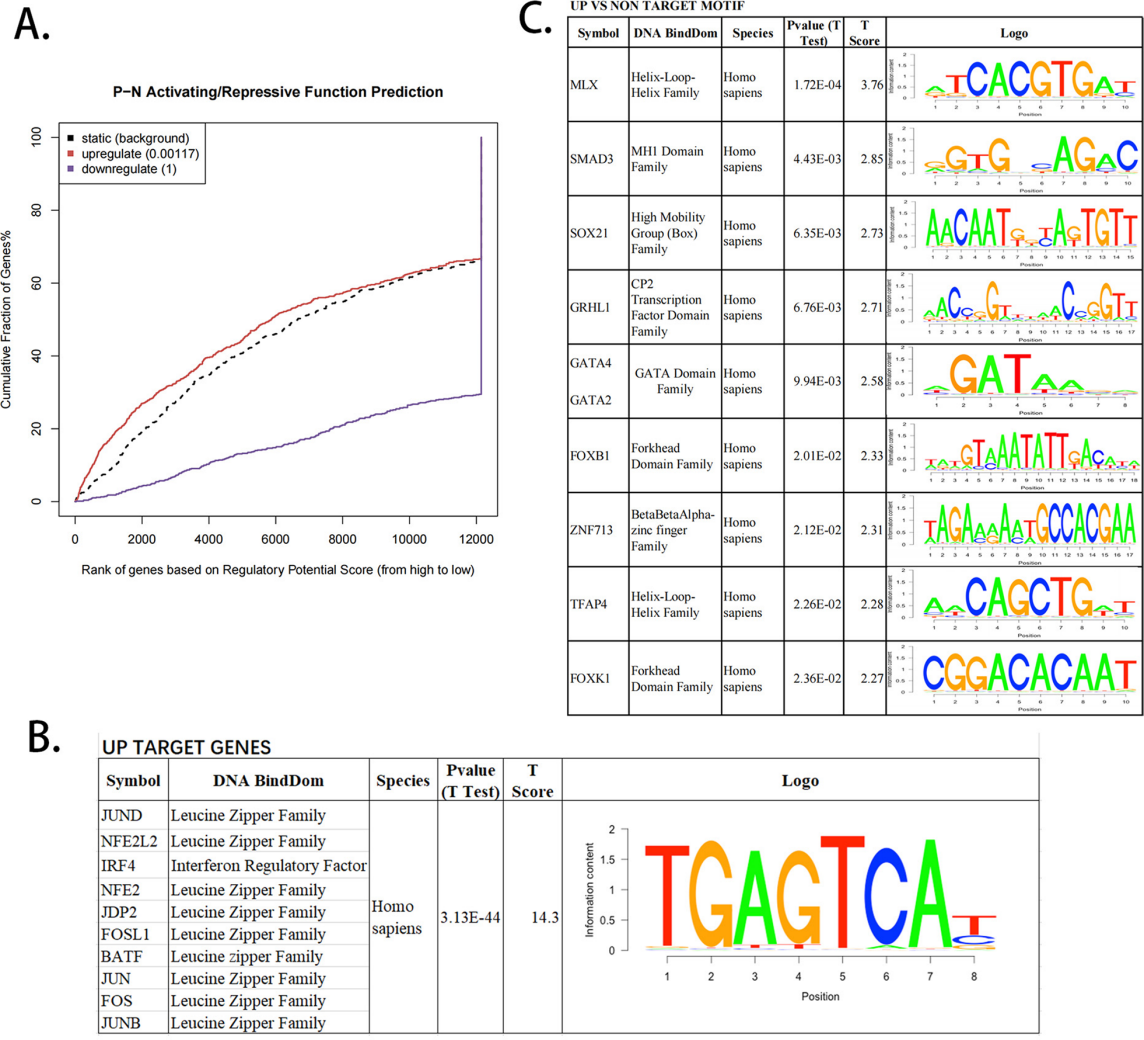
BETA activating/repressive function prediction of psoriatic lesions. **A** BETA analysis graphs depicting the effect of differentially open ATAC peaks in psoriatic lesions. Genes were ranked from high to low according to the regulatory potential of the corresponding chromatin peak. Purple lines represent downregulated genes, while red lines represent upregulated genes. **B** BETA-plus conjoins factor binding and differential gene expression data to analyze sequence motifs in upregulated target genes (UP). Because of their high similarity scores, JUND, NFE2L2, IRF4, and seven other Leucine Zipper family members are categorized into one group. **C** Motif comparison between UP and NON regions represent potential collaborating factors

Based on our RNA-seq and ATAC-seq data, we used BETA-plus to integrate differentially expressed genes and open accessible peaks [9, 15]. BETA identified 408 upregulated genes (rank product < 0.01, Additional file 6: Table S1) and 133 downregulated genes (Ratio_up/down_ = 3.1). We noticed that the cumulative fraction of upregulated genes was much higher than static background states (*P* = 0.001, Fig. 1A), but the downregulated genes were insignificant. We thus focused on the 408 genes in the following analysis. Of the 408 significant upregulated genes, 57 were directly targeted by transcription factor AP-1.

Chromatin accessibility can be reflected by other epigenetic markers, such as DNA methylation, histone modifications, and DNase hypersensitivity. To further annotate 408 genes with accessibility peaks, we looked at whether these peaks can be interpreted by other epigenetic modifications. Based on our genome-wide DNA methylation dataset previously generated from the same samples, we extracted methylation levels for loci located within the accessible peaks [9]. For the 18 methylation loci located within 408 peaks, we found all accessible peaks marked by hypomethylation compared with normal controls (Additional file 7: Table S2). Meanwhile, some open accessibility could be confirmed by active histone modification and DNase hypersensitivity, retrieved from UCSC database, such as OSMR, CDCP1, and SHB (Additional file 1: Figure S1A–C). These findings indicated that some psoriasis-associated open accessible peaks were linked with other types of epigenetic markers, but most of them could not be directly interpreted.

For 408 upregulated genes, we found that the accessible peaks contained a consensus binding sequence, mainly targeted by transcription factor AP-1 member JUND, NFE2L2, NFE2, JDP2, FOSL1, BATF, JUN, FOS, and JUNB (Fig. 1B). In addition, compared with nontargeted genes, motifs analysis found potentially collaborating factors: MLX, SMAD3 (t test, P < 0.01, Fig. 1C). The primary AP-1 protein families in mammalian cells are JUN and FOS, forming heterodimer and homodimer through their leucine-zipper domains [16]. Moreover, a variety of pathways by AP-1 mediate the inflammatory response of psoriasis, such as TNF-α and IL-17 [17]. In summary, the BETA algorithm helped us identify several AP-1 targeted genes that might be instrumental for psoriasis development.

### KEGG pathway analysis with upregulated target genes

**Fig. 2:**
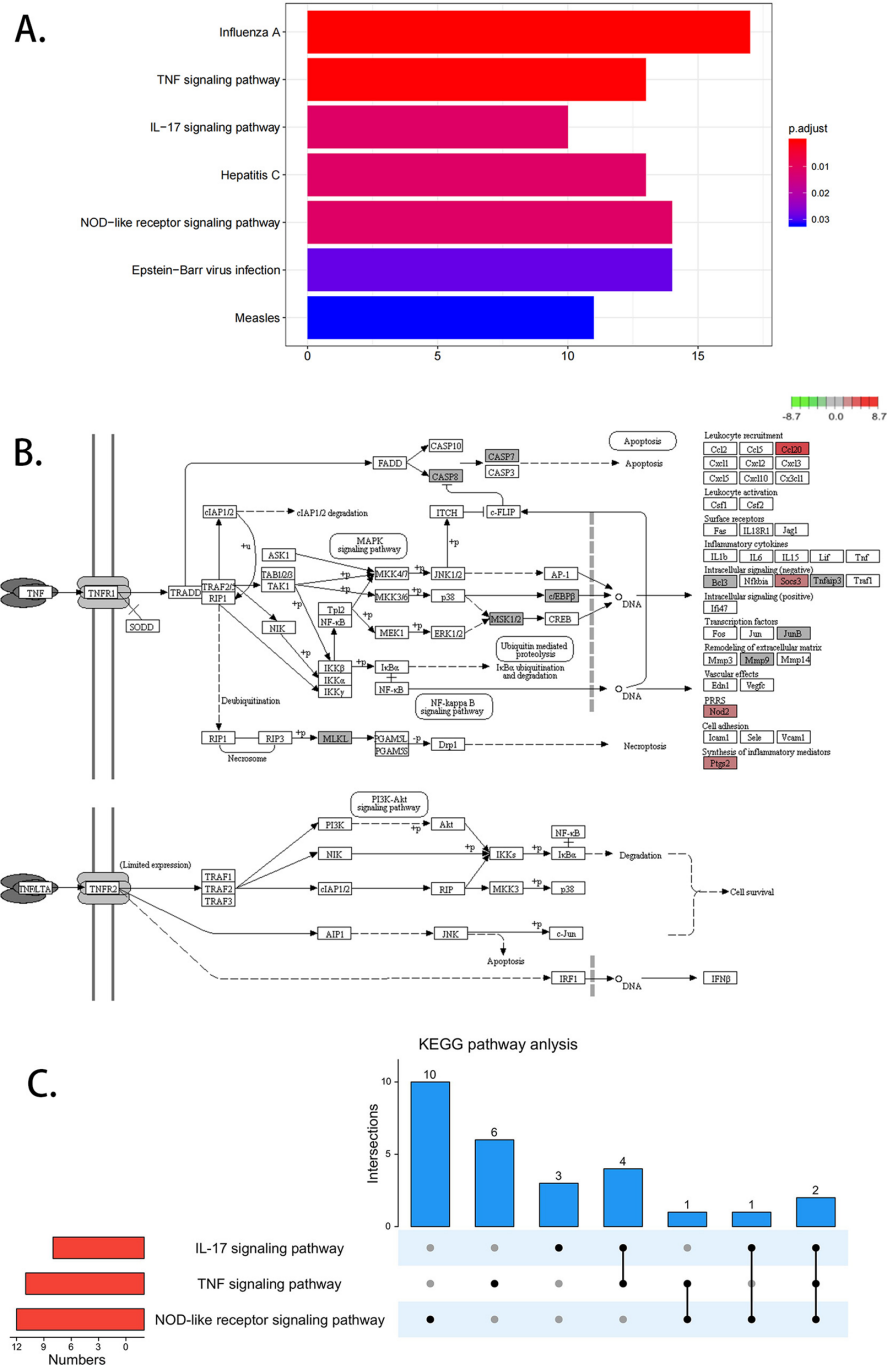
KEGG pathway analysis with upregulated target genes. **A** KEGG pathway analysis shows upregulated target genes enriched in Influenza A, tumor necrosis factor (TNF) signaling pathway, IL-17 signaling pathway (*P* < 0.05). **B** TNF signaling pathway diagram. Red represents upregulated genes, and the darker the color, the more significant it is. **C** Crosstalk of upregulated genes enriched in TNF signal pathway, IL-17 signal pathway, and NON-like receptor signal pathway

To further explore the functional mechanism of the 408 upregulated genes, we utilized KEGG pathway analysis. These genes were significantly enriched in Influenza A, TNF signaling pathway, IL-17 signaling pathway, Hepatitis C, NOD-like receptor signaling pathway, Epstein-Barr virus infection, and Measles (*P* < 0.05, Fig. 2A). TNF-mediated chronic inflammation by controlling innate and adaptive immune cells and induced diverse chronic inflammatory diseases, such as psoriasis [18]. Genes enriched in the TNF signaling pathway included NOD2, BCL3, SOCS3, CASP8, RPS6KA4, CCL20, JUNB, TNFAIP3, MLKL, MMP9, PTGS2, CASP7, CEBPB (Fig. 2B, Additional file 8: Table S3).

Furthermore, we found that the upregulated genes were enriched on IL-17 signaling pathway and NOD-like receptor signaling pathway, which were tightly related to psoriasis (Fig. 2A, Additional file 2: Figure S2A, B). The genes enriched in the IL-17 pathway include S100A7A, LCN2, CASP8, CCL20, TBK1, TNFAIP3, MAPK6, MMP9, PTGS2, and CEBPB (Additional file 8: Table S3). And the NOD-like receptor signaling pathway contains NOD2, IFI16, CASP8, OAS2, NLRX1, OAS3, TBK1, GBP5, TNFAIP3, MYD88, NAMPT, PANX1, OAS1, and PYDC1 (Additional file 8: Table S3). Meanwhile, the upregulated genes also enriched in some virus-related signaling pathway, such as Influenza A, Hepatitis C, Epstein-Barr virus infection, and Measles, suggesting that virus infection might be an important triggering factor for psoriasis development [5]. Moreover, these enriched signaling pathways also intersect (Fig. 2C). We found that CASP8 and TNFAIP3 were enriched in all three TNF, IL-17, and NOD-like receptor signaling pathway. MMP9, PTGS2, and CEBPB were implicated within both TNF and IL-17 signaling pathway. While TBK1 was settled in IL-17 and NOD-like receptor signaling pathway.

For the 133 downregulated genes, we found these genes could be targeted by AP-1 factors JUND, BATF, NFE2, JDP2, FOSL1, FOS, JUN, JUNB, and were enriched in “Adipocytokine signaling pathway,” “FoxO signaling pathway,” “AMPK signaling pathway” and some others (Additional file 3: Figure S3A–B).

### Target genes are associated with psoriasis histopathology

**Fig. 3:**
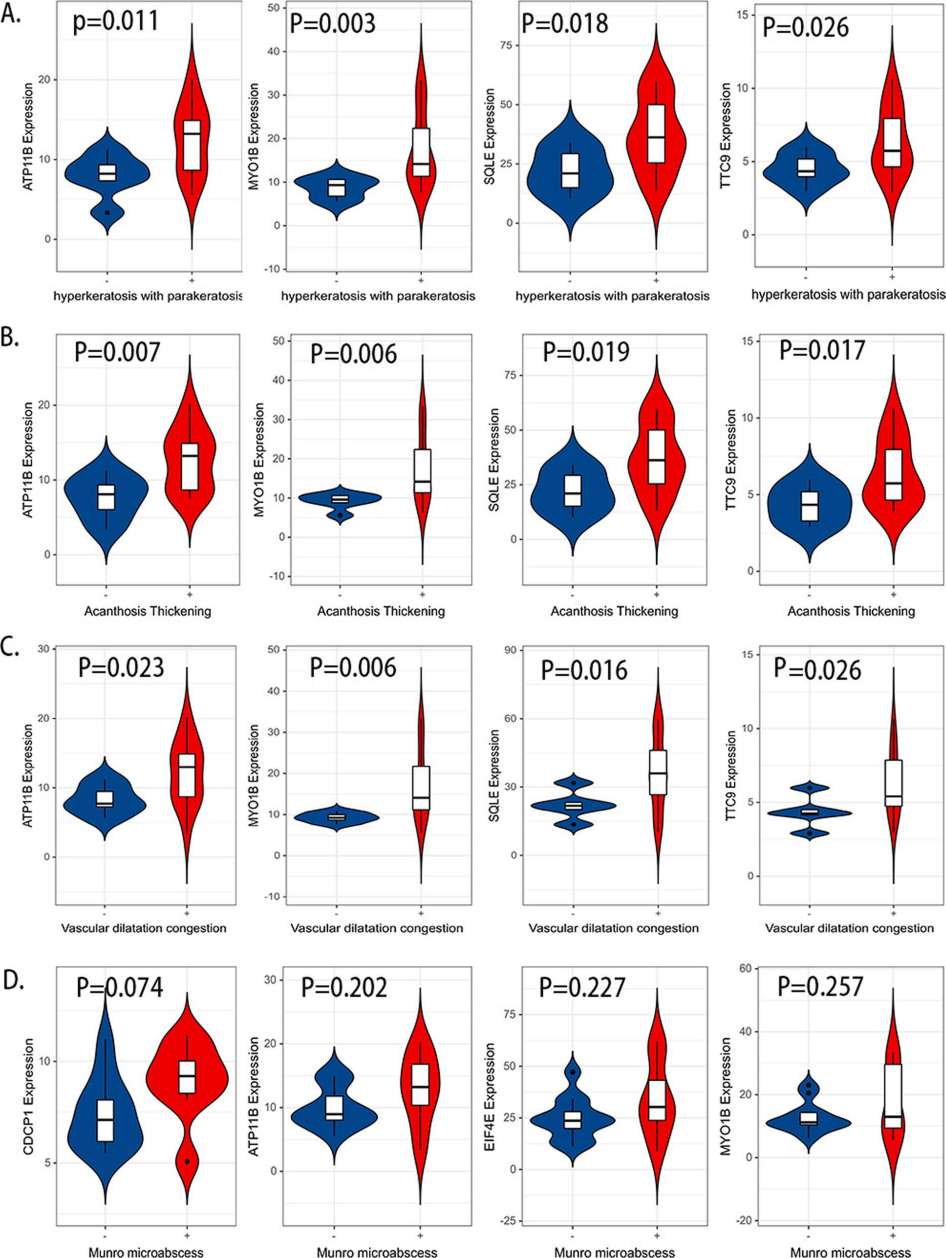
Altered expression of AP-1 targets in psoriatic lesions. Alteration expression of AP-1 targets in hyperkeratosis with parakeratosis (**A**), parakeratosis, acanthosis thickening (**B**), vascular dilatation congestion (**C**), and Munro micro-abscess (**D**)

The histopathological traits are important for psoriasis diagnosis and may vary among patients. To check whether histopathological traits be controlled by different genes, we analyzed all samples by pathological sections and grouped samples into positive and negative status for each pathological feature, including hyperkeratosis and parakeratosis, Munro micro-abscess, acanthosis thickening, vascular dilatation congestion, elongation of rete pegs, granulosa thinning, and lymphocyte infiltration. By retrieving our transcriptome sequencing data of 20 psoriatic skins, we compared expression differences of positive and negative status for each feature. We found that histopathological features can be modulated by different AP-1 targets. For example, expression difference of ATP11B, EIF4E, SQLE, MYO1B, RAP2B, TTC9, HECTD1, STRN, SDR9C7, RAB7A, LIMK2, FRMD6, and CLPX can be detected in hyperkeratosis with parakeratosis (Fig. 3A, Additional file 4: Figure S4A). The expression of these 13 genes also differed in patients with acanthosis thickening (Fig. 3B, Additional file 4: Figure S4B). In addition, the expression of TNFAIP3, ATP11B, MYO1B, SQLE, TTC9, EIF4E, RAP2B, and HECTD1 was increased in vascular dilatation congestion (Fig. 3C, Additional file 5: Figure S5A). The differential genes showed strong overlap, indicating a close link between the three features in psoriasis lesions.

No expression differences were associated with Munro micro-abscess, elongation of rete pegs, or granulosa thinning. Nevertheless, a trend can be perceived that the expression of CDCP1, ATP11B, MYO1B, and EIF4E increases in patients with Munro micro-abscess (Fig. 3D). While in patients with elongation of rete pegs, the expression of PPARD, MID1IP1, LDLR, and ID1 tend to be augmented (Additional file 5: Figure S5B). In patients with granulosa thinning, the expression of NUP210, TNFAIP3, CDCP1, and CEBPG tends to increase (Additional file 5: Figure S5C).

Meanwhile, we evaluated the relationships between several confounder factors (Age, PASI, BMI, Gender, Smoking) and the expressions of genes targeted by AP-1. Among 235 events (47 genes × 5 factors), it was found that 93.19% (219/235) had no statistical significance (*P* > 0.05, Additional file 9: Table S4). Most important, the genes correlated with confounder factors were not associated with pathological traits mentioned above, suggesting that these genes might not contribute to psoriasis histopathology.

### SQLE, STRN, EIF4E, and MYO1B might drive the development of psoriasis

To verify the reliability of 408 upregulated genes in our BETA analysis, we retrieved the publicly accessible expression array dataset with the largest psoriatic and control skin sample size (GSE30999). Among 408 genes in GSE30999, we found 91.42% (373/408) showed significant upregulation (*P* < 0.01), and 59.80% (244/408) suggested strong statistical significance with both *P* < 0.01 and |logFC|> 1, indicating that most of the upregulated genes can be confirmed by other datasets.

**Fig. 4:**
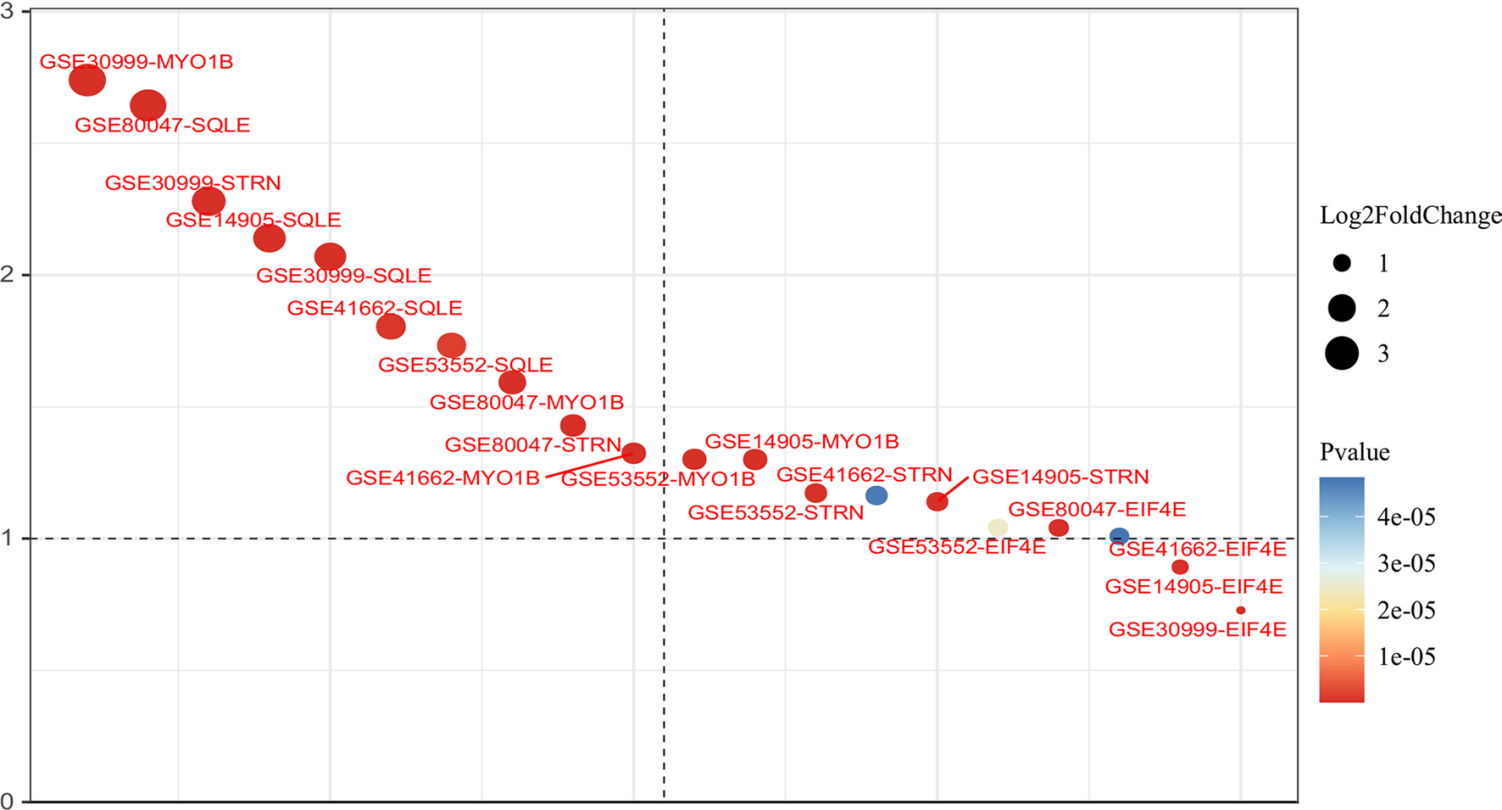
Expression significance of SQLE, STRN, EIF4E, and MYO1B genes in public databases. *Y*-axis: logFC of the expression difference in each GEO dataset (GSE80047, GSE53552, GSE41662, GSE30999, GSE14905)

To further explore the potential pathogenesis of psoriasis regulated by AP-1-mediated targets, we mined and analyzed the differential expression data in the GEO database (GSE80047, GSE53552, GSE41662, GSE30999, GSE14905), all expression data were generated from psoriatic and normal skins. Among 57 AP-1 targeted genes, SQLE, STRN, EIF4E, and MYO1B were consistently upregulated in five public datasets and our BETA analysis (*P* < 0.01, logFC > 1, Fig. 4), suggesting that AP-1 might modulate disease development through these four genes.

## Discussion

Psoriasis is a complex immune inflammatory disease. Moreover, the specific regulatory network is not precise. Here, applying BETA to integrate chromatin accessibility and transcription data, we found that AP-1 targets potentially affected the occurrence and development of psoriatic lesions.

BETA is an efficient tool for predicting target genes of TFs based on the relationship between binding sites and transcriptional expression [14]. And it calculates a potential regulatory score based on the number of peaks in a fixed window (100 kb by default) around each gene TSS and ranks the genes based on this score. Originally, BETA was designed for integrative analysis of ChIP-Seq and RNA-seq to predict particular TF target genes and whether the TF activates or inhibits these genes. However, for ATAC-seq, the signal is the chromatin-accessible region of the whole genome. ChIP-seq data stems from interference by a particular TF signal, while ATAC-seq comes from a broad range of TF bindings. We speculated that the openly accessible region of chromatin led to the alteration of nearby target gene expression and further induced the development of psoriatic.

Our findings implicated various aspects of the occurrence and development, including the dysfunction of keratinocyte proliferation and apoptosis, the destruction of the skin barrier, abnormal immune inflammatory response. Among 57 AP-1 targets, KLHDC7B, CDCP1, ID1, E2F8, EIF4E, CALML3, RAP2B, MYO1B are all associated with cell enhanced proliferation, especially in cancer [19–28]. These genes presented a novel possibility in the abnormal proliferation of psoriasis facilitated by AP-1. The expression of DDX3X in psoriatic lesions was significantly higher. DDX3X is a central decision maker in the formation of NLRP3 inflammasomes [29], and NLRP3 plays a vital role in inflammatory skin diseases, including psoriasis [30, 31]. It is probably that AP-1 binding induces DDX3X expression, further activates NLRP3 inflammasomes to mediate psoriasis, and leads to lymphocyte infiltration in psoriatic lesions. Paradoxically, DDX3X, LYN, and SESN2 upregulation have the opposite effect on NLRP3 inflammasome; DDX3X and LYN can activate, while SESN2 inhibits NLRP3 inflammasome [29, 32–34]. This phenomenon can be explained by the bias caused by the insufficient sample size in our study. Alternatively, AP-1 might play a two-sided function in psoriasis; when the activation of NLRP3 is superior to the inhibition, the pathogenic NLRP3 inflammasome is produced.

TNF and IL-17 signaling pathways are critical to the pathogenesis of psoriatic disease, and their biological agents have been widely used in disease treatment [35]. Our findings showed that the upregulated targets are enriched in TNF, IL-17, and NOD-like receptor signaling pathways. Furthermore, the three pathways interact with each other. TNFAIP3 is a common gene among TNF, IL-17, and NOD-like receptor signaling pathways. Previous studies have shown that the expression of TNFAIP3 is downregulated in non-lesional areas of psoriasis and further downregulated in lesional areas [36, 37]. However, our results show that the expression of TNFAIP3 decreased in psoriatic non-lesions, but there is no significant difference between psoriatic lesions and normal healthy skin. Since most of our samples are mild to moderate psoriasis vulgaris, some studies have shown that the expression level of TNFAIP3 is negatively correlated with the severity of the disease [36, 37]. We tentatively speculate that the level of TNFAIP3 expression targeted by AP-1 is a dynamic fluctuation in the occurrence and development of psoriasis. Interestingly, CASP8 was also enriched in all three pathways. CASP8 is an apical caspase protease that mediates both pro-death and pro-survival functions [38, 39]. CASP8 upregulates TNFAIP3 expression to promote PD-L1 ubiquitination and degradation [40]. Moreover, depression of PD-L1 may induce psoriasis [41, 42]. Afterward, MMP9, PTGS2, and CEBPB were enriched in TNF and IL-17 signaling pathways. It is reported that MMP9 may decrease PD-L1 expression by activating TGF-β [43]. PTGS2 and CEBPB are also related to PD-L1 regulation [44, 45]. These findings suggest AP-1 plays a diverse role in modulating TNF, IL-17, and NOD-like receptor signaling pathways.

Surprisingly, KEGG analysis suggested that upregulated genes were enriched in virus-related pathways, Influenza A, Hepatitis C, Epstein-Barr virus infection, and Measles. Viruses or external pathogens might attack the body via innate and, or adaptive immune responses [46]. As reported, Influenza vaccination can induce psoriasis [47]. Hepatitis C and psoriasis are closely related [48]. And EB virus injection increases the sensibility of various autoimmune diseases, such as multiple sclerosis and inflammatory bowel diseases, via CD8+ T-cell deficiency [49, 50]. An autoimmune response in psoriasis results from the interaction between innate and adaptive immune responses [51]. Hence, our KEGG analysis of 408 upregulated genes hints at the correlation between psoriasis and virus infection. And, 408 upregulated genes are associated with AP-1, so we speculate that AP-1 has a specific interaction in the relationship between viral infection and psoriasis. Actually, a recent publication revealed JUNB/AP-1 controlled the immune cell and microbiota interaction in mouse skins, partially reflecting our hypothesis [52].

AP-1 target genes might modulate different histopathological features. Patients with hyperkeratosis and parakeratosis, acanthosis thickening had increased expression of 13 the same genes, including SQLE, STRN, EIF4E, and MYO1B. Notably, SQLE, EIF4E, and MYO1B expression were increased in vascular dilatation congestion. As we know, the imbalance of keratinocytes in psoriasis includes hyperkeratosis with parakeratosis and acanthosis thickening, and the disorder of immune system will lead to the abnormal distribution of lymphocytes. Abnormalities of keratinocytes or immune system are a decisive part of the drive of psoriasis [53]. In this regard, it is interesting to note that AP-1 upregulates the gene expression of SQLE, STRN, EIF4E, and MYO1B might to promote the abnormality of keratinocytes and immune system to mediate the occurrence of psoriasis. Some studies demonstrated that the SQLE gene promotes the proliferation, invasion, and metastasis of diversified cells and the epithelial-to-mesenchymal transition (EMT) [54]. STRN participates in the progress of hepatocellular carcinoma by inducing EMT [55]. In addition, EIF4E is indispensable in TGFβ-induced EMT [56]. Overexpression of the EIF4E brings about improved translation of mRNAs encoding proteins refers to cell cycle control, proliferation, apoptosis, and angiogenesis [24]. Here we provide results that an increased expression of EIF4E in psoriasis is consistent with a previous experiment [24]. During EMT process of psoriatic keratinocytes, IL-17A, IL-13, and TGF-β are imperative regulators [57]. Importantly, IL-17A promotes the proliferation of epidermal keratinocytes [58]. Therefore, we speculate that AP-1 upregulates the expression of SQLE, STRN, EIF4E, and MYO1B; it brings keratinocytes EMT, which further mediates hyperkeratosis with parakeratosis and acanthosis thickening. Meanwhile, abnormal keratinocytes disrupt the epidermal immune network, especially IL-23/IL-17A-Th17 axis, an intertwined biological process [53]. In summary, we can speculate that AP-1 may also connect keratinocytes and the immune system interaction network to maintain the progression of psoriasis.

Nowadays, the therapeutic biological agents of psoriasis usually target the core pathway: IL-23/IL-17A-Th17 axis [51, 58, 59]. It consists of numerous proinflammatory cytokines and chemokine interactions, including IL-17, TNF- α [58, 59]. Considering AP-1 targets several critical genes involved in IL-17 and TNF signaling pathways, we have sufficient confidence to speculate that AP-1 has excellent potential to be a new therapeutic target for the treatment of psoriasis through regulating the skin immune microenvironment and proliferation of epidermal keratinocytes.

## Materials and methods

### ATAC-seq and transcriptome sequencing data

The ATAC-seq and transcriptome sequencing data were from two previous studies, and detailed information of enrolled samples has been described [9, 15]. Briefly, patients were based on the following criteria: (i) at least one well-demarcated, erythematous, scaly lesion verified by two dermatologists; (ii) confirmation of each lesion-bearing tissue by clinical histopathology; (iii) no systemic anti-psoriatic treatment for 2 weeks before skin biopsy; and (iv) no topical anti-psoriatic treatment for 1 week before the biopsy. All tissues were cut from a defined skin area and were immediately flash-frozen in liquid nitrogen. We summarized samples' main demographic and clinical characteristics from RNA-seq and ATAC-seq, including age, BMI, gender, smoking, and ethnicity (Additional file 10: Table S5). Pearson correlation coefficient was used to evaluate the continuous variables (age, BMI, PASI), and a chi-square test was used to assess the dichotomous variable (gender). Most of the smoking status in control was missing, so we could not calculate this point currently.

For transcriptome sequencing, a total of 60 skin tissues (20 lesional, 20 non-lesional, and 20 controls) were entered into the project, with raw data generated from an Illumina HiSeq 2500 Sequencer. On average, we obtained 72 ± 42 million reads per sample and an average mapping rate 93% when aligned and mapping to the human reference genome hg19. Cufflinks software calculates gene expression values fragments per kb per million (FPKM). And the differential expression test was performed by R package limma (version: 3.40.2). For ATAC-seq, the chromatin of o skins was processed for Tn5-mediated segmentation and adapter incorporation according to the manufacturer’s protocol, and raw data were generated from BGI-500 sequencer (BGI, Shenzhen, China). After alignment, mapping, and deduplication of the mitochondrial genome, accessible peaks were called by MACS2. The differential accessibility was evaluated by the R package edgeR. All enrolled samples were collected at the First Affiliated Hospital of Anhui Medical University, and all individuals signed the written informed consent under the Anhui Medical University-approved protocol.

### BETA analysis

BETA (version 1.0.7) is an exposed source at http://customer.org/ap/ [14]. Utilized the information of binding site and differential expression, BETA predicts the function of activation or inhibition of TFs, infers the target genes, and identifies the motif and its binders of TFs. All BETA calculations are based on the Regulatory Potential Scores of each target gene, which is the likelihood that a gene is regulated by a factor, and each gene estimates its regulatory potential. The regulatory potential is calculated as *S*_g_ = ${\sum }_{i=1}^{k}{e}^{-(0.5+4\Delta i)}$ [14, 60]. *k* is all binding sites near the TSS of gene (g) within the specified range (default 100 kb) of the different peaks. Δ is the exact distance between *k* and TSS, proportional to 100 KB, Δ = 0.1 means exact distance = 10 kb. The possibility of gene regulation by factors depends on the number of binding sites in the TSS region and the distance between the binding site and TSS. Data input takes a set of peaks as BED (tissue-specific open chromatin regions from ATAC-seq), and the differential gene expression results from RNA-seq. Analysis was worked using default parameters on the Galaxy Cistrome platform, apart from the significance threshold p < 0.05.

### KEGG pathway analysis

Kyoto Encyclopedia of Genes and Genomes (KEGG) pathway enrichment analysis, annotation, visualization was carried out using the R (https://www.r-project.org/) package clusterProfiler. The enriched pathway was plotted by package Pathview.

### Histopathological section

The collection of skin tissues came from the Department of Dermatology, the First Affiliated Hospital, Anhui Medical University. The overall histopathological abnormalities, including rete peg elongation, the presence of hyperkeratosis with parakeratosis, parakeratosis, Munro micro-abscess, elongation of rete pegs, focal hypergranulosis, granulosa thinning, acanthosis thickening, vascular dilatation congestion, and lymphocyte infiltration, were evaluated by H&E-stained slides. Finally, all the histopathological changes were collected in the form of presence or absence.

### Statistical analysis

GSE80047, GSE53552, GSE41662, GSE30999, and GSE14905 expression data matrix was downloaded from GEO database (https://www.ncbi.nlm.nih.gov/geo/). Limma package (version: 3.40.2) was used to identify the differentially expressed genes. All statistical analyses were performed on the R (version 4.1.1) platform. T test, Pearson correlation coefficient, and chi-square test were used to evaluate the significance via SPSS. *P* < 0.05 was considered statistically significant.

## Supplementary Information

**Additional file 1. Figure S1: **Accessible peaks and their correspondingepigenetic changes. The yellow band at the yellow arrow is the accessiblepeaks annotated to OSMR (A), CDCP1 (B), and SHB (C); the methylationstatus of parallel peak regions decreased significantly.

**Additional file 2. Figure S2: **KEGG pathway analysis with upregulatedtarget genes. (A): IL-17 signaling pathway diagram. (B): NON-like receptorsignaling pathway diagram. Red represents upregulated genes, and thedarker the color, the more significant it is.

**Additional file 3. Figure S3: **BETA and KEGG pathway analysis withdownregulated target genes. (A): Motifs in downregulated target genes(DOWN) yield by BETA. Because of their high similarity scores, JUND, IRF4,BATF, and six other Leucine Zipper family members are categorized intoone group. (B): KEGG pathway analysis with downregulated target genes,and shows downregulated target genes enriched in “Adipocytokinesignaling pathway,” “FoxO signaling pathway,” “AMPK signaling pathway,”and some others (P < 0.05).

**Additional file 4. Figure S4: **Altered expression of AP-1 targets in psoriaticlesions. Altered expression of AP-1 targets in lymphocytes infiltration(A), Elongation of rete pegs (B), Granulosa thinning (C), and vasculardilatation congestion (D).

**Additional file 5. Figure S5: **Alteration expression of AP-1 targets inpsoriatic lesions. Altered expression of AP-1 targets in hyperkeratosis withparakeratosis (A) and acanthosis thickening (B).

**Additional file 6: Table S1.** BETA finding for 408 upregulated genes and expression profiles from our RNA-seq and GSE30999.

**Additional file 7: Table S2.** Methylation levels for loci located within the accessible peaks.

**Additional file 8: Table S3.** KEGG pathway analysis shows upregulated target genes enriched in TNF, NON-like receptor, and IL-17 signaling pathway.

**Additional file 9: Table S4.** Correlation between confounders and expressions of 46 AP-1 targeted genes. Between Age, PASI, BMI, and gene expression are assessed using the Pearson correlation coefficient, and the P values of gender-related and smoking-related gene expression are calculated by t test, and P < 0.05 means statistical significance.

**Additional file 10: Table S5.** The main demographic and clinical characteristics of samples. Pearson correlation coefficient was used to evaluate the continuous variables (age, BMI), and a chi-square test was used to assess the dichotomous variable (Gender). *P* < 0.05 means statistical significance.

## Data Availability

All data generated or analyzed during this study are publicly accessed; ATAC-seq (PRJNA597655) and RNA-seq (PRJNA686863).

## Abbreviations

ATAC-seq: Assay for targeting accessible-chromatin with high-throughput sequencing
BETA: Binding and expression target analysis
TF: Transcription factor
KEGG: Kyoto Encyclopedia of Genes and Genomes
TNF: Tumor necrosis factor
NOD: Nucleotide-binding oligomerization domain containing
IL-17: Interleukin 17
AP-1: Activator Protein 1
SQLE: Squalene epoxidase
STRN: Striatin
EIF4E: Eukaryotic translation initiation factor 4E
MYO1B: Myosin IB
IL-23: Interleukin 23
FRA1: FOSL1, Fos-related antigen 1, FOS Like 1
OSMR: Oncostatin M receptor
CDCP1: CUB domain-containing protein 1
SHB: SH2 domain-containing adaptor protein B
JDP2: Jun dimerization protein 2
NFE2L2: Nuclear factor erythroid-derived 2-like 2
IRF4: Interferon regulatory factor 4
BATF: Basic leucine zipper ATF-like transcription factor
MLX: MAX dimerization protein
SMAD3: SMAD family member 3
BCL3: B cell CLL/lymphoma 3
SOCS3: Suppressor of cytokine signaling 3
CASP8: Caspase 8
RPS6KA4: Ribosomal protein S6 kinase A4
CCL20: C-C motif chemokine ligand 20
TNFAIP3: TNF alpha-induced protein 3
MLKL: Mixed lineage kinase domain-like pseudokinase
MMP9: Matrix metallopeptidase 9
PTGS2: Prostaglandin-endoperoxide synthase 2
CASP7: Caspase 7
CEBPB: CCAAT enhancer-binding protein beta
S100A7A: S100 calcium-binding protein A7A
LCN2: Lipocalin 2
TBK1: TANK-binding kinase 1
MAPK6: Mitogen-activated protein kinase 6
IFI16: Interferon gamma-inducible protein 16
OAS2: 2'-5'-Oligoadenylate synthetase 2
NLRX1: NLR family member X1
OAS3: 2′-5′-Oligoadenylate synthetase 3
GBP5: Guanylate-binding protein 5
MYD88: MYD88 innate immune signal transduction adaptor
NAMPT: Nicotinamide phosphoribosyltransferase
PANX1: Pannexin 1
OAS1: 2′-5′-Oligoadenylate synthetase 1
PYDC1: Pyrin domain containing 1
ATP11B: ATPase phospholipid transporting 11B
RAP2B: RAP2B, member of RAS oncogene family
TTC9: Tetratricopeptide repeat domain 9
HECTD1: HECT domain E3 ubiquitin protein ligase 1
SDR9C7: Short chain dehydrogenase/reductase family 9C, member 7
RAB7A: RAB7A, member RAS oncogene family
LIMK2: LIM domain kinase 2
FRMD6: FERM domain containing 6
CLPX: Caseinolytic mitochondrial matrix peptidase chaperone subunit
PPARD: Peroxisome proliferator-activated receptor delta
MID1IP1: MID1 interacting protein 1; LDLR: low-density lipoprotein receptor
ID1: Inhibitor of DNA binding 1, HLH protein
NUP210: Nucleoporin 210
CEBPG: CCAAT/enhancer-binding protein gamma
GEO: Gene Expression Omnibus
TSS: Transcription start site
EMT: Epithelial-to-mesenchymal transition. PRS: Potential regulatory score
DDX3X: DEAD-box helicase 3 X-linked
NLRP3: NACHT, LRR, and PYD domains-containing protein 3
SESN2: Sestrin-2
PD-L1: Programmed death-1
TGF-β: Transforming growth factor-β
PTGS2: Prostaglandin G/H synthase 2
Th17: T helper cell 17
PASI: Psoriasis area and severity index
BMI: Body mass index
