# Supplementary figures and images for: Chromatin accessibility and transcriptome integrative analysis revealed AP-1-mediated genes potentially modulate histopathology features in psoriasis
Source: Clin Epigenetics. 2022 Mar 11;14:38. doi: 10.1186/s13148-022-01250-6 (PMC8917665; doi:10.1186/s13148-022-01250-6)

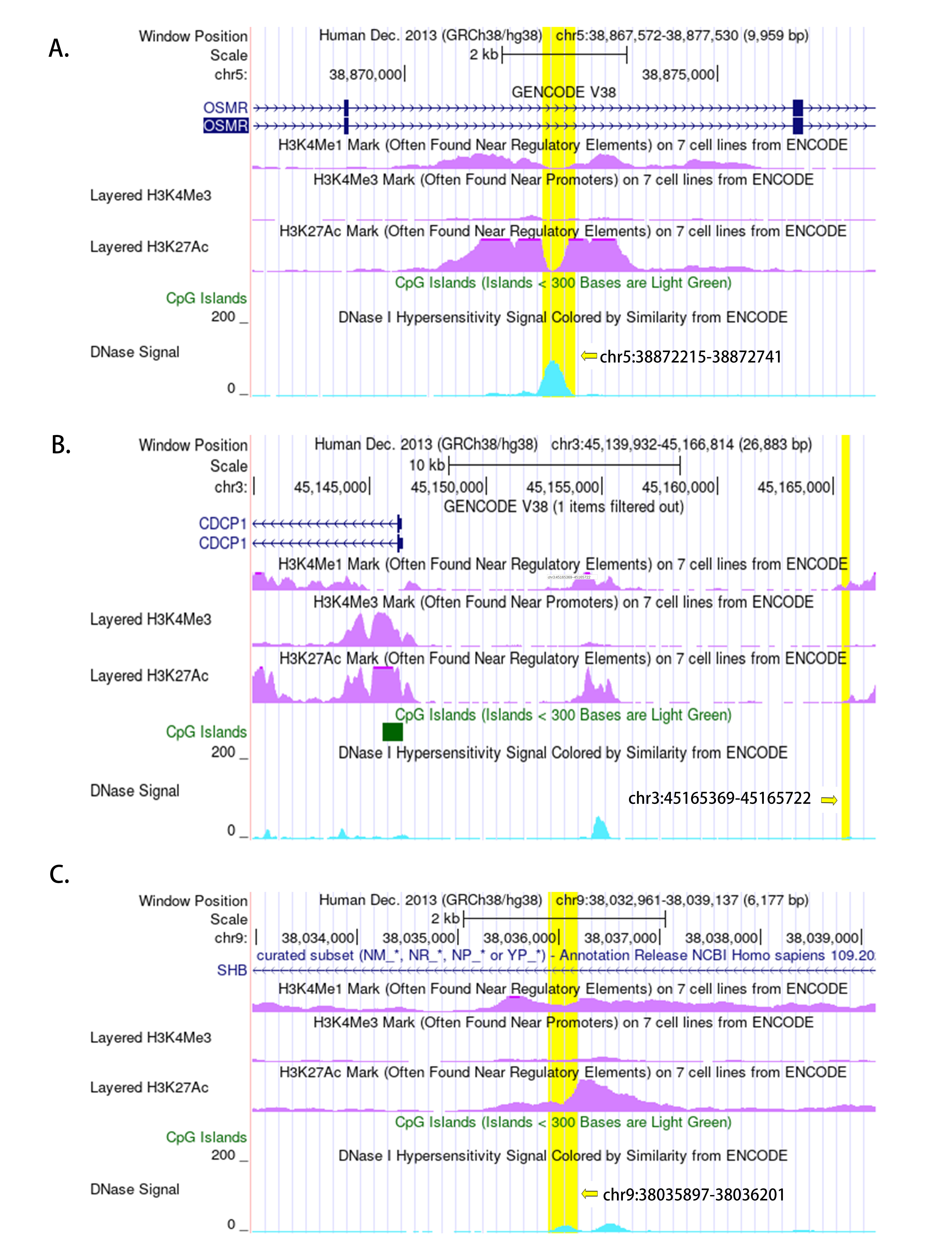

Supplement: Supplementary file 1 — Additional file 1. Figure S1: Accessible peaks and their correspondingepigenetic changes. The yellow band at the yellow arrow is the accessiblepeaks annotated to OSMR (A), CDCP1 (B), and SHB (C); the methylationstatus of parallel peak regions decreased significantly. [file 13148_2022_1250_MOESM1_ESM.jpg]

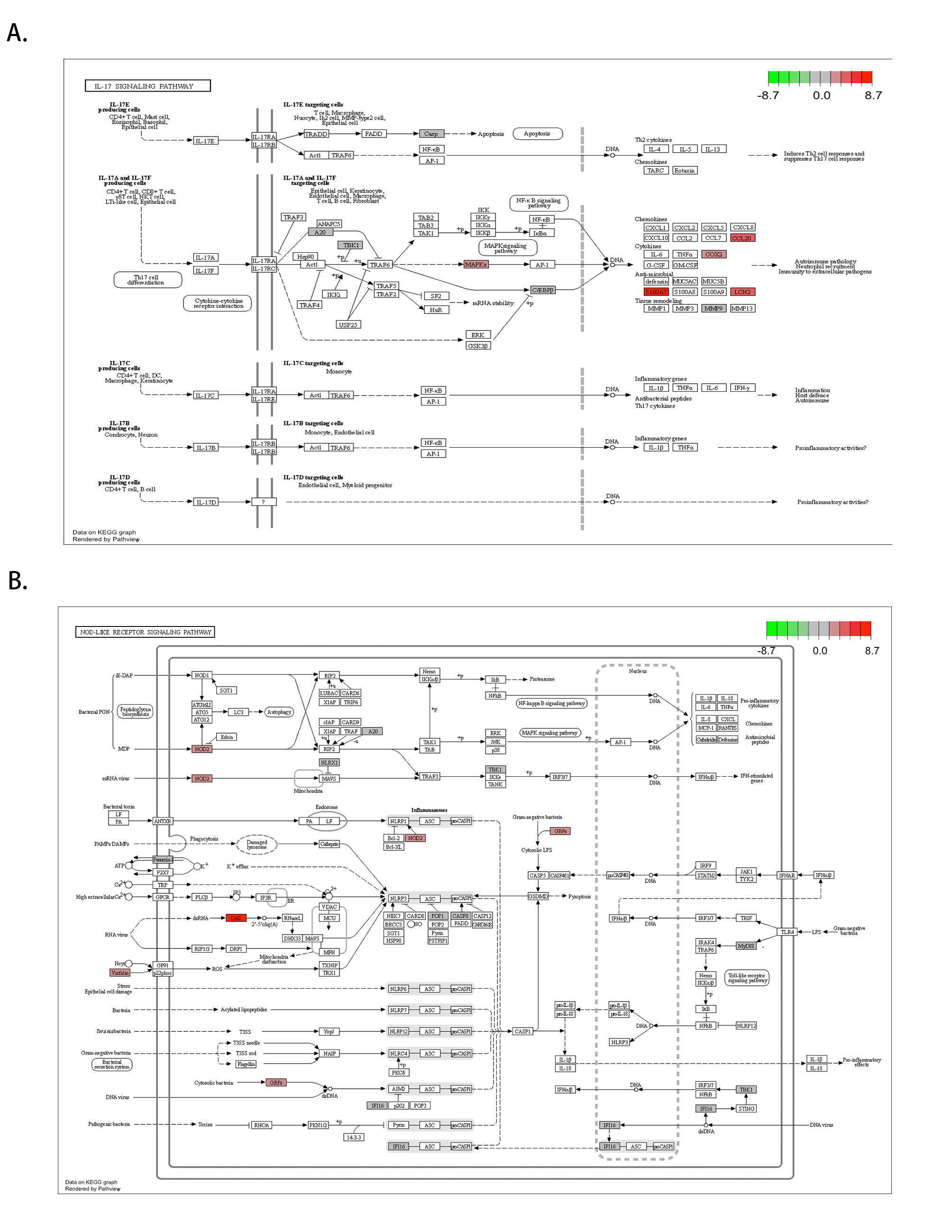

Supplement: Supplementary file 2 — Additional file 2. Figure S2: KEGG pathway analysis with upregulatedtarget genes. (A): IL-17 signaling pathway diagram. (B): NON-like receptorsignaling pathway diagram. Red represents upregulated genes, and thedarker the color, the more significant it is. [file 13148_2022_1250_MOESM2_ESM.jpg]

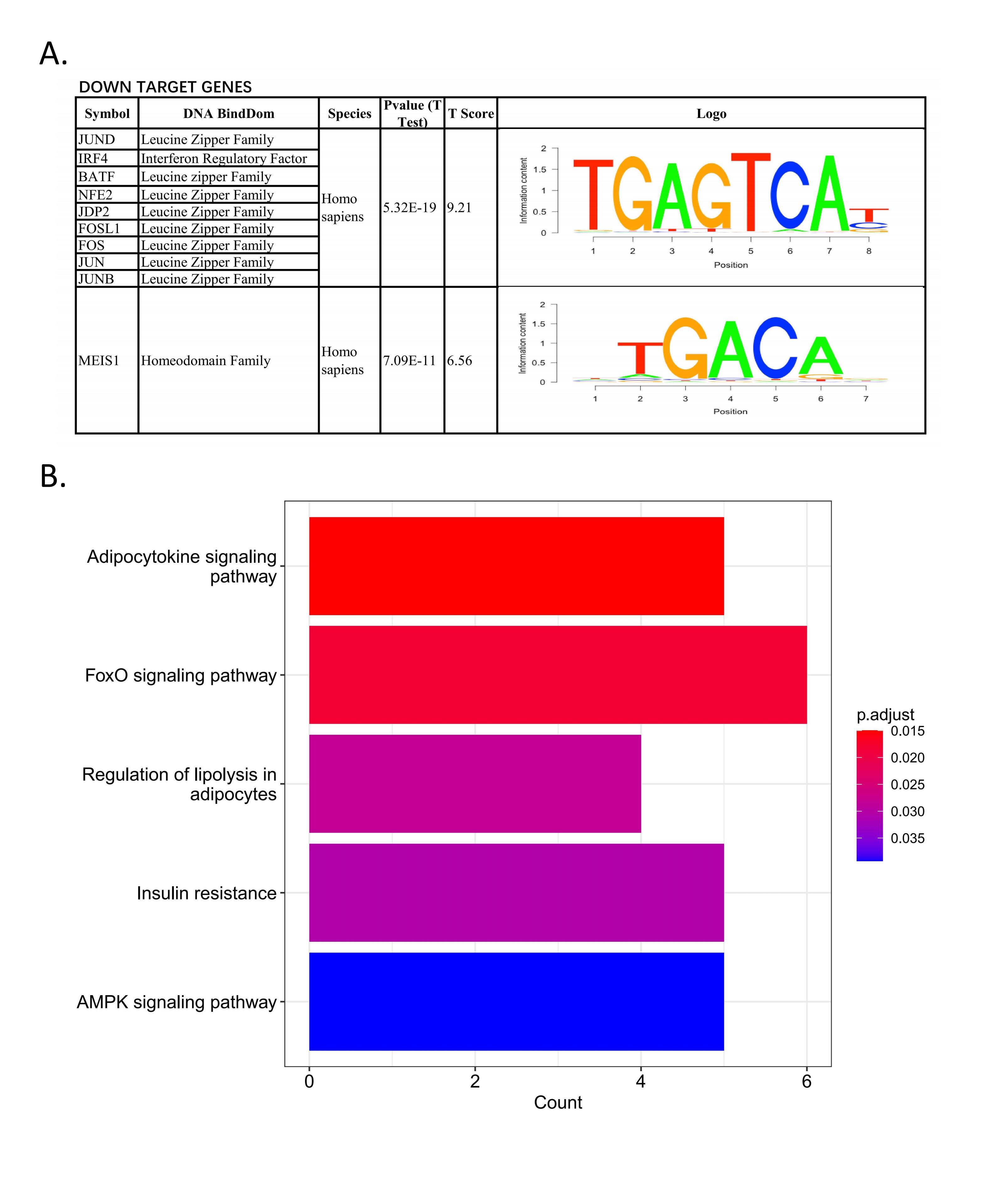

Supplement: Supplementary file 3 — Additional file 3. Figure S3: BETA and KEGG pathway analysis withdownregulated target genes. (A): Motifs in downregulated target genes(DOWN) yield by BETA. Because of their high similarity scores, JUND, IRF4,BATF, and six other Leucine Zipper family members are categorized intoone group. (B): KEGG pathway analysis with downregulated target genes,and shows downregulated target genes enriched in “Adipocytokinesignaling pathway,” “FoxO signaling pathway,” “AMPK signaling pathway,”and some others (P < 0.05). [file 13148_2022_1250_MOESM3_ESM.jpg]

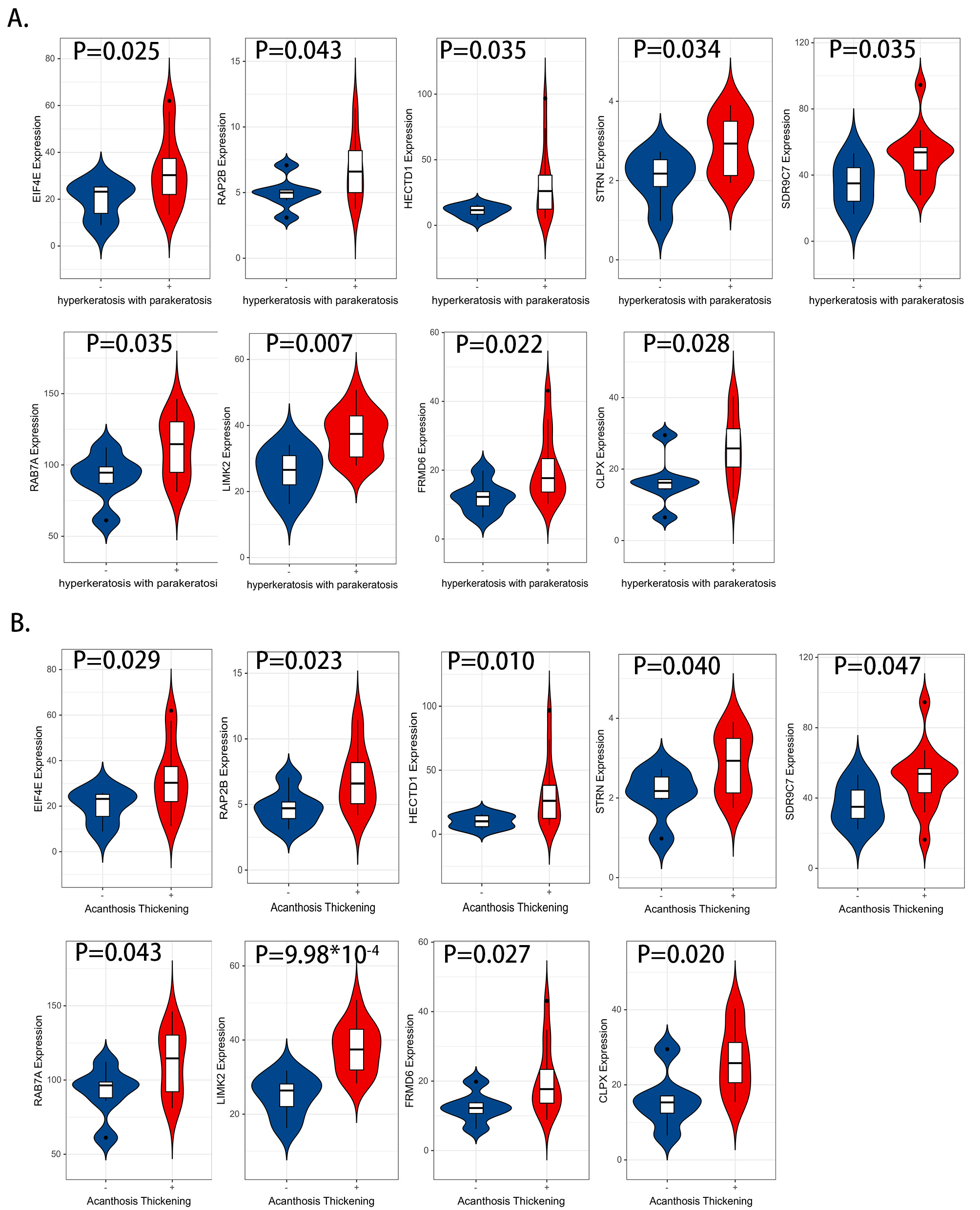

Supplement: Supplementary file 4 — Additional file 4. Figure S4: Altered expression of AP-1 targets in psoriaticlesions. Altered expression of AP-1 targets in lymphocytes infiltration(A), Elongation of rete pegs (B), Granulosa thinning (C), and vasculardilatation congestion (D). [file 13148_2022_1250_MOESM4_ESM.jpg]

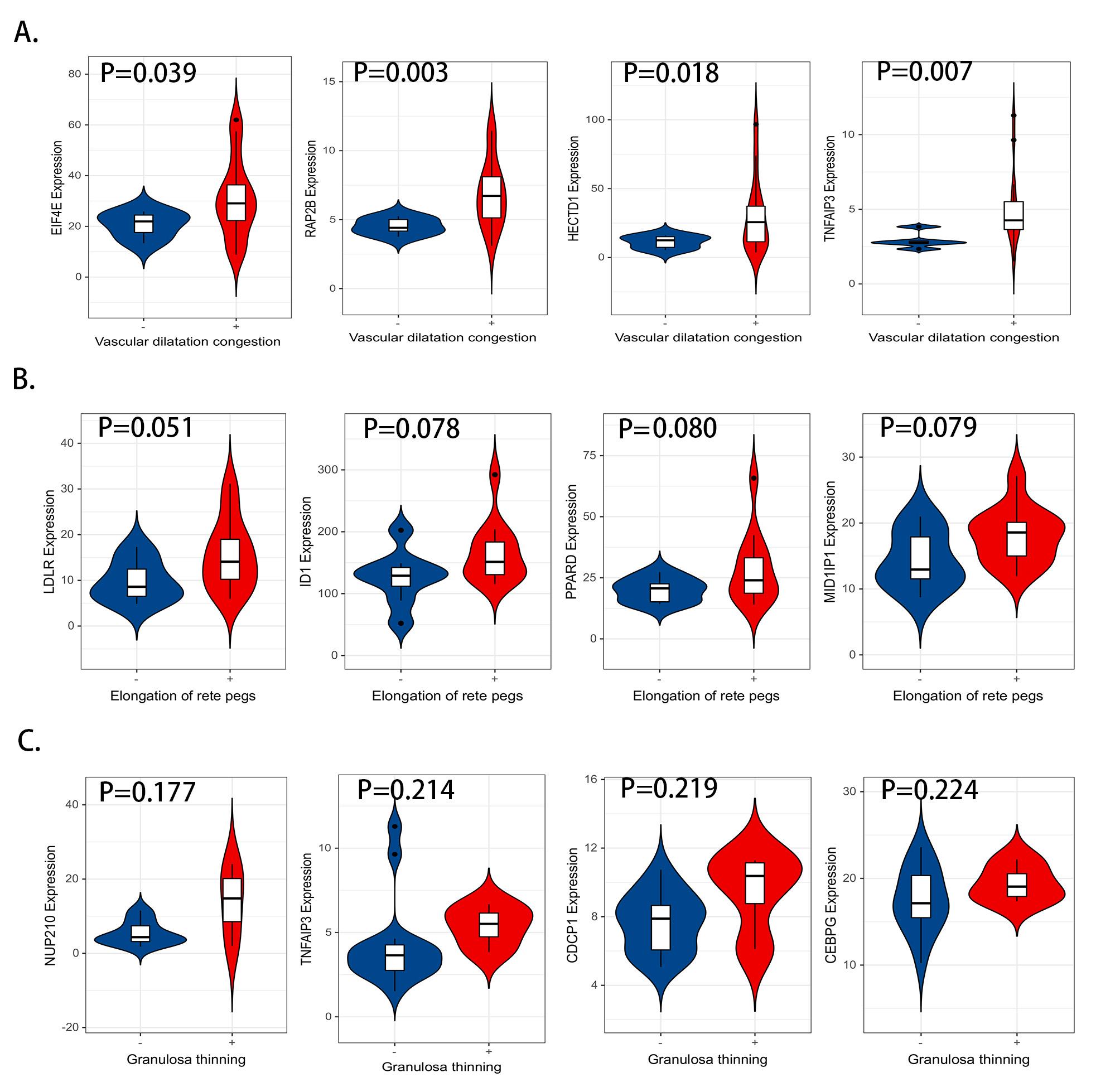

Supplement: Supplementary file 5 — Additional file 5. Figure S5: Alteration expression of AP-1 targets inpsoriatic lesions. Altered expression of AP-1 targets in hyperkeratosis withparakeratosis (A) and acanthosis thickening (B). [file 13148_2022_1250_MOESM5_ESM.jpg]
